# Evaluation of the impact of refined nursing care on schizophrenia patients

**DOI:** 10.1097/MD.0000000000040848

**Published:** 2025-01-17

**Authors:** Yuanling Zhang, Wenbin Liang, Jiao Gui

**Affiliations:** a Department of PICU2, Wudong Hospital of Wuhan, Wuhan, Hubei, China.

**Keywords:** cognitive function, medication adherence, quality of life, refined nursing, schizophrenia

## Abstract

This study evaluates the impact of refined nursing interventions on patients with schizophrenia, focusing on disease severity, cognitive function, medication adherence, quality of life, and medication-related complications. The aim is to provide evidence for enhancing future clinical treatments. From January 2022 to January 2024, 201 schizophrenia patients were enrolled based on specific inclusion and exclusion criteria. Patients were allocated into 2 groups using propensity score matching: an experimental group that received refined nursing care and a control group that received routine care. Outcome measures included Positive and Negative Syndrome Scale scores, cognitive assessments (Mini-Mental State Examination and Nurses’ Observation Scale for Inpatient Evaluation), medication adherence, quality of life, relapse rates, and medication-related side effects. After propensity score matching, baseline characteristics of the experimental and control groups were comparable. Following nursing interventions, the experimental group demonstrated significant improvements in Positive and Negative Syndrome Scale scores, cognitive assessments (Mini-Mental State Examination and Nurses’ Observation Scale for Inpatient Evaluation), medication adherence, and quality of life compared to the control group. The experimental group also showed lower relapse rates and fewer medication side effects, confirming the enhanced effectiveness of refined nursing interventions. Refined nursing care significantly improved disease outcomes, cognitive function, medication adherence, and quality of life in schizophrenia patients, while reducing relapse rates and medication-related complications, highlighting its clinical value in psychiatric care.

## 1. Introduction

Schizophrenia is a chronic mental disorder caused by a combination of genetic and environmental factors. Its primary features include disturbances in perception, emotion, thought, and cognitive abilities. The global prevalence of schizophrenia is approximately 0.4%, while in China, it has risen from 0.27% in the 1950s to 1.347% in 2016.^[1,2]^ In China, schizophrenia patients make up two-thirds of all mental illness cases, leading to significant familial and socioeconomic burdens, with annual medical costs reaching $15,500 to $22,300, and even higher for patients with relapse or disability.^[3]^

Schizophrenia is characterized by its long course, persistence, and high relapse rate, severely impacting patients’ mental health and quality of life.^[4]^ During treatment, most patients experience feelings of inferiority, lose confidence in recovery, lead a sedentary lifestyle, and even become socially withdrawn. With advances in psychiatry, controlling symptoms and preventing relapse are no longer the ultimate goals of treatment.^[5]^ Improving the quality of life and facilitating social reintegration are now also essential. High-quality, comprehensive care provided by psychiatric nurses during the recovery phase is crucial for enhancing clinical outcomes, improving prognosis, and quality of life.

Refined nursing is a model based on medical science and combined with humanistic care. Its core involves a comprehensive assessment of patients’ physiological, psychological, and social needs, leading to individualized care interventions.^[6]^ Compared to traditional nursing models, refined nursing places greater emphasis on the details and accuracy of each care process, from initial assessment upon admission to the entire treatment process and follow-up care.^[7]^ This model relies on the latest research findings, tailored to the specific disease type, treatment outcomes, and rehabilitation potential of the patient, aiming to improve treatment efficacy while enhancing patient comfort and satisfaction with care. Originating from the “refined management” theory, this model emphasizes improving care quality and efficiency while embodying a patient-centered approach. It has been widely applied in public health events, care management, and patient examinations, showing significant results in reducing hospital stays, accelerating recovery, and optimizing care outcomes.^[6]^

However, research on the application of refined nursing interventions in schizophrenia and their potential impacts is limited. This study aims to explore the benefits of refined nursing interventions for schizophrenia patients by comparing aspects such as disease severity, psychological and physiological states, functional activities, quality of life, and medication complications, to provide insights for future clinical treatment.

## 2. Methods

### 2.1. Study design

This study was approved by the Ethics Committee of Wudong Hospital of Wuhan. This study is a retrospective analysis of schizophrenia patients who visited our hospital from January 2020 to January 2022. The full study period, plus follow-up for the next 2 years. The full study dates are January 2020 to January 2024. A total of 201 valid cases were included based on inclusion and exclusion criteria. Patients were divided into 2 groups according to the type of nursing they previously received: the experimental group (93 patients) who had received refined nursing and the control group (108 patients) who had received routine nursing.

Inclusion criteria: diagnosis of schizophrenia according to the Chinese Classification and Diagnostic Criteria for Mental Disorders. Age >18 years. Previous receipt of complete routine or refined nursing. Complete electronic medical records and follow-up information. Informed consent obtained from the patients and their families.

Exclusion criteria: acute mental disorders or severe somatic or organic brain diseases. Other psychiatric disorders affecting cognition. Intellectual disabilities preventing assessment. Incomplete receipt of routine or refined nursing. Incomplete historical information.

### 2.2. Nursing methods

The control group received routine nursing interventions, including secondary care as per medical instructions. Nurses were responsible for daily living care, providing lifestyle guidance, and communicating with family members to enhance family support and emotional encouragement. The nursing process included maintaining effective scientific communication, guiding patients in healthy eating, emphasizing personal hygiene and cleanliness, regularly changing clothes, and ensuring proper ventilation. Patients were required to adhere strictly to medication instructions and receive care in a clean and tidy ward environment to promote recovery.

Refined nursing for schizophrenia management included the following components: (1) cognitive behavioral nursing: conducting small educational sessions to inform patients about schizophrenia. The education was delivered in a simple, understandable manner, explaining the disease progression, treatment, and important behaviors for self-care. Providing guidance on daily activities, especially healthy behaviors and habits during the recovery phase. This included supervising daily activities such as eating and medication adherence, correcting maladaptive behavior patterns, improving disease awareness among patients and their families, encouraging timely medical consultation, and enhancing treatment compliance. (2) Psychological care: engaging in active communication to offer psychological support, alleviate anxiety and unfamiliarity, and build a trusting relationship. Implementing targeted psychological interventions, such as playing soothing music daily to relieve agitation, helping patients maintain a stable mood, boosting recovery confidence, and facilitating social reintegration. (3) Functional rehabilitation exercises: developing personalized rehabilitation training plans based on individual patient conditions and preferences. Encouraging participation in small games and recreational activities. (4) Social and family support: encouraging family members to provide emotional support and establishing a social support system to enhance patients’ treatment adherence and improve quality of life. (5) Dietary and nutritional care: creating individualized dietary plans based on patient preferences, promoting the intake of high-fiber and vitamin-rich foods, and increasing the consumption of fats, proteins, and sugars. Encouraging appropriate outdoor exercise to strengthen physical health and facilitate mental relaxation.

### 2.3. Propensity score matching

As this study is a retrospective cohort study, it is necessary to match patients’ baseline information to reduce the impact of differences on subsequent outcome comparisons. Propensity score matching^[8]^ is a statistical method used to reduce the influence of confounding variables in observational studies. It calculates the probability of each individual receiving treatment or control conditions, thereby balancing baseline characteristics between treatment and control groups. This allows for a more accurate estimation of treatment effects. We matched the following variables between the experimental and control groups: age, gender, duration of illness, cognitive scores, Positive and Negative Syndrome Scale (PANSS) scores, behavioral observation scores, schizophrenia subtype, medication adherence, education level, and medical history. The fitting of the propensity score model for different nursing methods was assessed using the Hosmer–Lemeshow test.^[9]^

### 2.4. Observation indicators

#### 2.4.1. Quality of life score

The World Health Organization Quality of Life-BREF^[10]^ is a tool used to assess an individual’s quality of life. It includes 26 items covering 4 domains: physical health, psychological health, social relationships, and environment.

#### 2.4.2. Cognitive function score

The Mini-Mental State Examination (MMSE)^[11]^ is a brief cognitive screening tool. It assesses orientation, memory, attention, calculation, language, and visual-spatial abilities. The total score is out of 30, with lower scores indicating more severe cognitive impairment. Scores are categorized as follows: 0 to 9 (severe cognitive impairment), 10 to 20 (moderate cognitive impairment), 21 to 26 (mild cognitive impairment), and 27 to 30 (normal).

#### 2.4.3. Nurses’ Observation Scale for Inpatient Evaluation (NOSIE)^[12]^

The NOSIE is a tool for assessing the behavior and symptoms of psychiatric inpatients. It helps nurses record and analyze patients’ conditions in an inpatient setting, focusing primarily on behavioral disturbances in psychiatric patients. The scale consists of 30 items across 7 dimensions. It is a frequency scale, with items scored from 0 to 4 based on the frequency of specific symptoms, where a higher total score indicates a better condition. The scale has demonstrated reliability and validity in psychiatric patients, with a Cronbach alpha coefficient of 0.6902 and a split-half reliability of 0.8682.

#### 2.4.4. Medication adherence

Medication adherence prior to hospitalization was assessed using Arango method, which involves inquiring with family members and patients about adherence over the past year. Adherence was categorized into 3 levels: high adherence (10–12 months), moderate adherence (5–9 months), and low adherence (<4 months).^[13]^

During hospitalization, medication adherence was primarily measured through reports from family members and patients, with pill counts used as a supplementary estimate. The adherence level was assessed according to definitions by Zhu Yinglu and Li Zheng,^[14]^ and categorized as follows:

High adherence: actively accepting medication during hospitalization and continuing to adhere to the medication regimen as prescribed after discharge.

Moderate adherence: passively accepting medication during hospitalization but failing to adhere to the prescribed regimen after discharge.

Low adherence: refusing medication during hospitalization and continuing to refuse medication after discharge.

#### 2.4.5. Insight and Treatment Attitudes Questionnaire (ITAQ)

The ITAQ^[15]^ is used to evaluate patients’ self-awareness of their illness and their attitudes toward treatment. It consists of 11 items covering illness insight and treatment attitudes, scored from 0 to 2. A score of 2 indicates complete insight, 1 indicates partial insight, and 0 indicates no insight. The total score ranges from 0 to 22, with higher scores reflecting deeper understanding of the illness, a more positive attitude toward treatment, and greater completeness of insight.

#### 2.4.6. Medication side effect assessment

The Treatment Emergent Symptom Scale^[16]^ is a tool for assessing adverse symptoms or side effects occurring during treatment. It is designed to help clinicians and researchers identify and quantify treatment-related side effects. Key features of Treatment Emergent Symptom Scale include symptom assessment, severity rating, frequency, and duration.

#### 2.4.7. PANSS score

The PANSS^[17]^ is used to evaluate symptoms in schizophrenia patients. It consists of 30 items divided into 3 sections: positive symptoms, negative symptoms, and general psychopathology. Each item is scored from 1 (absent) to 7 (severe), providing a comprehensive assessment of the severity of symptoms and clinical changes.

#### 2.4.8. Relapse criteria^[18]^

Relapse was defined by the following criteria: a >25% increase in the total PANSS score. A Clinical Global Impression^[19]^ score of ≥ 6 (markedly worse) on the efficacy rating (Clinical Global Impression-I). Severe self-injury or property destruction. Clear suicidal or homicidal ideation. Relapse was considered if any of the above criteria were met and persisted for more than 1 week.

### 2.5. Statistical analysis

Statistical analyses were conducted using SPSS software. For continuous variables, the Shapiro–Wilk test was first performed to assess the normality of the data distribution. If the data were normally distributed, results were presented as mean ± standard deviation and independent samples *t* tests were used for group comparisons. For non-normally distributed data, results were presented as median and interquartile range, and Kruskal–Wallis one-way analysis of variance was used for group comparisons. For categorical variables, data were presented as frequencies or percentages, and chi-square tests were used to analyze differences between groups. The level of statistical significance was set at *P* < .05. All statistical tests were two-sided.

## 3. Results

### 3.1. Matching of general information between different nursing methods

Propensity score matching was used to balance the baseline characteristics between the 2 groups in terms of age, gender, duration of illness, education level, cognitive scores (MMSE), PANSS scores, medication adherence, schizophrenia subtype, and medical history, as shown in Table 1. This process eliminated significant differences in variables such as age, duration of illness, MMSE, PANSS scores, schizophrenia subtype, and medical history between the experimental and control groups, ensuring that the baseline characteristics were comparable for subsequent comparisons.

**Table 1:** Matching results of basic information.

Variables	Before matching			After matching		
	Experimental group	Control group	*P*	Experimental group	Control group	*P*
Total number of individuals	93	108		72	72	
Age (years)	36.65 ± 4.71	32.16 ± 3.24	<.001	34.21 ± 2.01	33.89 ± 1.96	.32
Gender			.95			.13
Male	53	62		41	32	
Female	40	46		31	40	
Course of disease	8.23 ± 5.23	6.21 ± 4.36	.01	6.89 ± 3.16	6.71 ± 3.22	.77
Educational level			.19			.84
Junior high school or below	13	22		10	12	
Senior high school	41	53		29	30	
Senior high school above	39	33		33	30	
MMSE	24.26 ± 2.31	22.22 ± 3.12	<.001	24.37 ± 1.41	24.01 ± 1.32	.26
PANSS (total)	84.32 ± 6.11	88.36 ± 5.03	.001	83.26 ± 4.14	84.16 ± 3.43	.16
Drug compliance			.34			.13
High	21	32		14	22	
Moderate	43	51		36	25	
Low	29	25		22	25	
Classification of schizophrenia			.006			.61
Hebephrenic type	16	26		16	14	
Simple type	21	14		15	14	
Catatonic type	33	45		30	32	
Intolerance style	15	23		9	12	
Undifferentiated type	8	0		2	0	
Medical history						
Hypertension			.002			.80
Yes	33	61		31	30	
No	60	47		40	42	
Diabetes			.001			.48
Yes	24	51		24	28	
No	69	57		48	44	
Propensity score ($\bar{X}\pm S$)	0.52 ± 0.13		<.001	0.21 ± 0.07		.310

### 3.2. Impact of refined nursing on the severity of schizophrenia

The severity of schizophrenia was compared based on PANSS scores, which were assessed in 3 areas: positive symptoms, negative symptoms, and general psychopathology, as shown in Table 2. Prior to nursing interventions, there were no significant differences between the groups in negative symptoms, positive symptoms, or general psychopathology scores, as the PANSS total score was included in the propensity score matching to ensure similar disease severity between the groups. After nursing interventions, scores in all 3 areas decreased within each group compared to pre-intervention levels. Furthermore, post-intervention scores in the experimental group were significantly lower than those in the control group (*P* < .05).

**Table 2:** PANSS sub-score assessment of schizophrenia severity.

Group	Positive symptoms		Negative symptoms		General psychopathology symptoms	
	Before care	After care	Before care	After care	Before care	After care
n	72	72	72	72	72	72
Control group	29.16 ± 2.46	27.41 ± 2.12*	10.12 ± 1.21	7.46 ± 1.96*	45.63 ± 3.26	27.64 ± 4.16*
Experimental group	30.04 ± 2.33	25.23 ± 2.22*	10.45 ± 1.03	6.62 ± 1.47*	46.12 ± 2.68	22.56 ± 3.11*
T value	-1.12	2.20	-0.02	1.36	-0.13	4.26
*P*	.45	.01	.51	.02	.44	<.001

PANSS = Positive and Negative Syndrome Scale.

* It indicated that there was significant difference between the 2 groups (*P* < .05).

### 3.3. Impact of refined nursing on cognitive and behavioral abilities in schizophrenia patients

Cognitive function and adherence to treatment significantly impact disease recovery. We assessed cognitive and behavioral abilities using the MMSE and NOSIE, as shown in Table 3. There were no significant differences in MMSE and NOSIE levels between the 2 groups before nursing interventions. Post-intervention scores for both measures were significantly higher than pre-intervention scores, with the experimental group showing significantly better outcomes than the control group. After nursing interventions, the experimental group had MMSE and NOSIE scores of 25.87 and 184.33, respectively, compared to 24.67 and 172.36 in the control group.

**Table 3:** Cognitive score MMSE and behavioral ability NOSIE.

Group	MMSE		T value	*P*	NOSIE		T value	*P*
	Before care	After care			Before care	After care		
n	72	72			72	72		
Control group	23.41 ± 1.32	24.67 ± 1.79	-2.38	.02	167.12 ± 12.62	172.36 ± 14.02	-11.21	<.001
Experimental group	23.37 ± 1.41	25.87 ± 1.63	-5.85	<.001	171.01 ± 12.43	184.33 ± 14.32	-10.21	<.001
T value	0.21	-2.60			-4.23	-12.06		
*P*	.84	.01			.06	<.001		

MMSE = Mini-Mental State Examination, NOSIE = Nurses’ Observation Scale for Inpatient Evaluation.

### 3.4. Impact of refined nursing on insight, treatment attitudes, and medication adherence in schizophrenia patients

Research indicates that medication adherence in schizophrenia patients is generally poor. We assessed patients’ insight and recognition of their condition using the ITAQ and compared medication adherence between the groups. As shown in Table 4, both the experimental and control groups showed improvements in ITAQ scores, with the experimental group scoring significantly higher (18.26) compared to the control group (15.13). Regarding medication adherence, as shown in Table 5, the experimental group demonstrated significantly better adherence compared to the control group, with 32 cases of high adherence and 38 cases of moderate adherence.

**Table 4:** ITAQ comparison between the 2 groups.

Group	ITAQ		T value	*P*
	Before care	After care		
n	72	72		
Control group	6.32 ± 3.21	15.13 ± 4.65	-7.26	<.001
Experimental group	6.80 ± 3.41	18.26 ± 5.40	-10.26	<.001
T value	-0.63	-3.16		
*P*	.45	.02		

ITAQ = Insight and Treatment Attitudes Questionnaire.

**Table 5:** Comparison of drug adherence between the 2 groups.

Group	Before admission			After admission to hospital		
	High	Moderate	Low	High	Moderate	Low
Experimental group (n = 72)	14	36	22	32	38	2
Control group (n = 72)	22	25	25	25	32	15
X^2^	3.95			11.31		
*P*	.138			.003		

### 3.5. Impact of refined nursing on relapse rates and medication-related complications in schizophrenia patients

**Figure 1.**
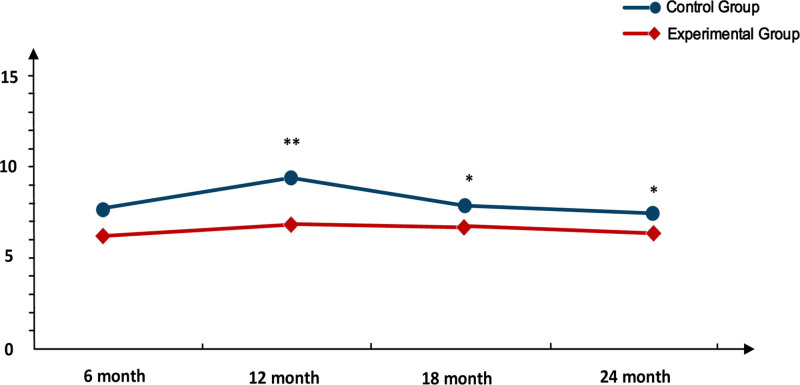
Recurrence rate after discharge.

Subsequently, we examined disease relapse rates and medication-related complications following improvements in medication adherence. As shown in Figure 1, there were no significant differences in relapse rates between the groups at 6 months post-discharge. However, relapse rates at 12, 18, and 24 months were significantly higher in the control group compared to the experimental group, with both groups experiencing a peak in relapses at 12 months. As indicated in Table 6, there were significant differences in medication side effects between the groups post-intervention, with 58 patients in the experimental group and 46 in the control group experiencing mild or no symptoms.

**Table 6:** Comparison of drug side effect score between the 2 groups.

Group	Before care		After care	
	Mild or no side effects	Serious side effect	Mild or no side effects	Serious side effect
Experimental group (n = 72)	34	38	58	14
Control group (n = 72)	44	28	46	26
X^2^	2.797		4.986	
*P*	.094		.025	

### 3.6. Impact of refined nursing on quality of life in schizophrenia patients

Finally, we assessed the quality of life of patients in both groups across 4 dimensions: physical health, psychological health, social relationships, and environment, as shown in Table 7. There were no significant differences in scores between the groups before the nursing interventions, but all scores significantly improved after the interventions. Notably, in the physical health, social relationships, and environment dimensions, the experimental group scored significantly higher than the control group. However, there were no significant differences between the groups in the psychological health dimension.

**Table 7:** Comparison of quality of life scores between the 2 groups.

Group	Physiological dimension		Psychological dimension		Social dimension		Environmental dimension	
	Before care	After care	Before care	After care	Before care	After care	Before care	After care
Experimental group (n = 72)	56.05 ± 4.12	62.21 ± 3.95*	37.02 ± 3.22	42.26 ± 2.65*	7.40 ± 1.21	8.65 ± 1.03*	60.60 ± 3.33	66.45 ± 2.15*
Control group (n = 72)	54.36 ± 5.31	57.44 ± 4.11*	36.64 ± 3.21	40.36 ± 2.34*	7.31 ± 1.11	7.69 ± 1.41*	59.45 ± 2.89	64.56 ± 2.11*
T value	2.16	5.11	1.01	1.25	0.68	-0.45	0.45	2.10
*P*	.312	.031	.546	.051	.211	.03	.312	.04

* It indicated that there was significant difference between the 2 groups (*P* < .05).

## 4. Discussion

Schizophrenia is a complex disorder characterized by prolonged duration and frequent relapse.^[20]^ Simple treatment is insufficient to improve the patient’s quality of life; rather, a combination of behavioral habits, psychological insight, and social support is crucial for long-term recovery.^[21]^ This study, balanced for general baseline information, explores the multifaceted benefits of refined nursing for schizophrenia patients.

Firstly, the impact of nursing approaches on schizophrenia symptoms was evaluated using PANSS scores. Refined nursing significantly improved negative symptoms, positive symptoms, and general psychopathology compared to standard care. This demonstrates a clear improvement in symptoms due to the refined nursing approach.

Subsequently, differences in cognitive and behavioral abilities were assessed. The refined nursing group showed significantly better outcomes than the control group, likely due to its comprehensive approach, which includes patient and family education and guidance on daily living habits.

Medication adherence is a critical, yet often overlooked, aspect of treatment. Adherence issues, whether during hospitalization or after discharge, significantly impact treatment effectiveness. Poor adherence increases relapse risk, adding to medical costs and severely affecting the quality of life for both patients and caregivers.^[22]^ Consistent with our findings, refined nursing improves patients’ insight into their condition and enhances medication adherence.

Research indicates that long-term medication can impact patients’ metabolic functions, potentially leading to diabetes and negatively affecting their quality of life.^[23]^ Therefore, merely increasing medication adherence is not enough; it is crucial to assess whether improved adherence reduces relapse rates and avoids medication-related complications. Our study found that refined nursing not only improved medication adherence but also effectively reduced disease relapse, particularly during the 12-month post-discharge peak period.^[24]^ Additionally, increased adherence under refined nursing did not result in additional medication side effects, likely due to the emphasis on disease and treatment knowledge.

Finally, we evaluated patients’ quality of life across physical, psychological, social, and environmental dimensions. Significant improvements were observed in the physical, social, and environmental dimensions for the refined nursing group compared to the control group, though no significant differences were found in the psychological dimension.

This study has some limitations. As a retrospective study, data collection was constrained by the completeness of records, and long-term follow-up information might be biased. Additionally, the sample size, due to matching of baseline information, limits subgroup analysis. Future research should involve larger sample sizes to address these limitations.

## 5. Conclusion

In schizophrenia patients, refined nursing interventions significantly outperform standard care in terms of disease severity, cognitive function, medication adherence, and quality of life. Moreover, this intervention reduces medication side effects and relapse rates, demonstrating a marked advantage in improving overall health and treatment outcomes.

## Author contributions

**Conceptualization:** Yuanling Zhang, Wenbin Liang, Jiao Gui.

**Data curation:** Yuanling Zhang, Wenbin Liang, Jiao Gui.

**Formal analysis:** Wenbin Liang, Jiao Gui.

**Funding acquisition:** Yuanling Zhang.

**Investigation:** Wenbin Liang, Jiao Gui.

**Methodology:** Wenbin Liang, Jiao Gui.

**Supervision:** Wenbin Liang, Jiao Gui.

**Validation:** Yuanling Zhang, Wenbin Liang.

**Writing – original draft:** Yuanling Zhang, Jiao Gui.

**Writing – review & editing:** Yuanling Zhang, Jiao Gui.
